# Low-level ^40^Ca determinations using nitrous oxide with reaction cell inductively coupled plasma–tandem mass spectrometry

**DOI:** 10.1007/s00216-022-04146-9

**Published:** 2022-05-31

**Authors:** Shaun T. Lancaster, Thomas Prohaska, Johanna Irrgeher

**Affiliations:** 1 Department of General, Analytical and Physical Chemistry, Chair of General and Analytical Chemistry, Montanuniversität Leoben, 8700 Leoben, Austria; 2 Department of Physics and Astronomy, University of Calgary, Calgary, Canada

**Keywords:** Calcium, ICP-MS/MS, N_2_O, Mass shift

## Abstract

In inductively coupled plasma mass spectrometry, the most abundant Ca isotope (^40^Ca) suffers from isobaric interference with argon, hindering the potential for low detection limits of Ca. A powerful approach is to remove the interference by using a reaction gas in a reaction cell. Ammonia (NH_3_) has proven to be an effective reaction gas by process of a charge transfer reaction. However, NH_3_ is highly corrosive and toxic and cannot remove isobaric ^40^ K. Therefore, this work proposes the use of nitrous oxide (N_2_O) to mass shift the target analyte ^40^Ca to ^40^Ca^16^O^+^ as a non-corrosive and non-toxic alternative. Instrument performance testing demonstrated that N_2_O was capable of reaching equivalent detection limits (0.015 ng g^−1^) and background equivalence concentrations (0.041 ng g^−1^) to that of NH_3_ and limited by the blank only. Further investigation of matrix interferences with synthetic standards highlighted that the N_2_O approach supports the separation of potassium (K) and magnesium (Mg)–based interferences at tested concentrations of more than 600 times and almost 800 times higher than Ca respectively, whereas NH_3_ was found to only support the removal of Mg. This work highlights a clear advantage of N_2_O for low-level Ca determinations with high matrix loads, as well as compatibility with other instrumentation sensitive to corrosion that supports reaction cell technology.

**Graphical abstract:**

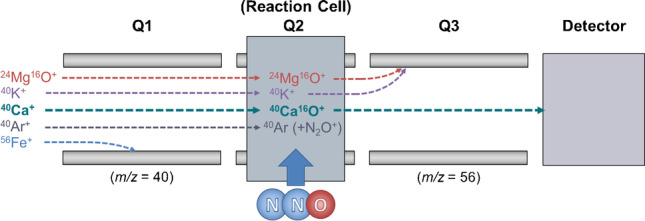

## Introduction

Inductively coupled plasma mass spectrometry (ICP-MS) is a widely used tool that boasts high sensitivity, low limit of detection (LOD), and high sample throughput. However, LODs of ICP-MS measurements can suffer due to spectral interferences from monatomic or polyatomic ions with the same mass to charge ratio (*m*/*z*). Calcium (Ca) is an example of an element that suffers from such interferences. In this case, the major isotope, ^40^Ca, which has a natural abundance of 96.941% [1], shares an isobaric interference with argon (Ar), which is used as both a carrier gas and for the generation of the plasma. To separate these, the required mass resolution would be > 190,000 (Table 1). As such, sensitivity and detection limits are greatly hampered by resorting to using less abundant Ca isotopes, such as ^42^Ca (0.647% abundance), ^43^Ca (0.135% abundance), and ^44^Ca (2.086% abundance) [1].

**Table 1:** List of interferences for Ca on *m*/*z* 40. Mass resolution was calculated from IUPAC Periodic Table of the Elements and Isotopes [1]. Negative resolutions indicate that the atomic mass of the interfering species is greater than that of.^40^Ca

Interference on ^40^Ca^+^	Abundance (%)	Atomic mass (u)	Required mass resolution
^40^Ca^+^	96.9	39.943	–
^40^Ar^+^	99.6	39.962	192,058
^40^ K^+^	0.0117	39.964	− 28,394
^24^Mg^16^O^+^	78.8	39.980	− 2302
^80^Se^++^	49.6	39.958	9229
^80^Kr^++^	2.29	39.958	9079
^39^K^1^H^+^	93.2	39.972	− 4471
^23^Na^17^O^+^	0.038	39.989	− 1520

One method commonly employed to overcome spectral interferences is the introduction of a reaction gas via a reaction cell. Recently developed instruments utilize an additional quadrupole as a mass filter in front of the reaction cell to avoid the formation of new interferences in the cell (ICP–tandem mass spectrometry (MS/MS)) [2, 3]. Ammonia (NH_3_) has been typically used as a reaction gas to remove interference of ^40^Ar^+^ for on-mass determination of ^40^Ca^+^ [4], as the charge transfer reaction (M^+^  + NH_3_ → NH_3_^+^  + M) occurs at a much higher rate for Ar^+^ than for Ca^+^ [2, 5, 6]. However, NH_3_ is a corrosive and toxic gas [7]. Therefore, it cannot be used in every instrument and its usage is sometimes limited in some laboratories. It is therefore of interest to assess suitable alternatives for greater sustainability regarding primarily the protection of the instrumentation.

Other reaction gases have also been used for on-mass determinations of ^40^Ca, such as methane [8, 9] and hydrogen [10]. However, mass-shift reactions of ^40^Ca using a reaction gas (e.g. ^40^Ca → ^40^Ca^16^O^+^) are less widely reported. Oxygen is the traditional reaction gas for analyte mass-shift determinations [2]. However, low formation of the CaO^+^ product ion renders this approach unfavourable [11]. Nitrous oxide (N_2_O), on the other hand, is a more reactive alternative to oxygen [12, 13] and has been explored recently for a number of elements using ICP-MS/MS systems [14–16], highlighting a broad scope for use in routine multi-element analysis. While N_2_O shows high reactivity, it is notably much less corrosive than NH_3_ and may serve as a suitable alternative. Apart from ^40^Ar, other interferences have to be considered if Ca is determined at low levels in a complex matrix (see Table 1).

While sensitivity can be enhanced and the LOD can be decreased by using cell methodology, it is important to note that current LODs are limited by background levels of Ca. Therefore, it is important to be highly considerate of sources of contamination. Wu et al. suggested the use of a clean laboratory environment can allow for lower detection limits [9]. Retzmann et al. described in detail how to minimize the Ca background and reported that, e.g., the use of nitrile gloves and clean-room wipes were major sources of Ca contamination and should be avoided [17].

This work aims to evaluate the novel usage of N_2_O for quantification of Ca at low levels in complex matrices by ICP-MS/MS using ^40^Ca. Optimization of both the cell gas flow rates and possible internal standards was carried out. Instrument performance parameters for both reaction gases (NH_3_ and N_2_O) were compared with each other, as well as with the determination of ^40^Ca under standard conditions (no cell gas). Additionally, the effectiveness of the removal of sample matrix interferences caused mainly by magnesium (Mg) and potassium (K) was also investigated for each cell gas.

## Materials and methods

All preparations and measurements were made in a clean room (ISO class 8) to minimize the risks of contamination. Polyethylene gloves (Carl Roth GmbH, Karlsruhe, Germany) were used on top of nitrile gloves to avoid Ca contamination. The use of clean-room wipes and paper towels were avoided throughout.

### Chemicals and standards

Nitric acid (*w* = 65%, p.a. grade; Carl Roth GmbH) was first purified by sub-boiling using a sub-boiling distillation system (Savillex DST-4000, AHF Analysentechnik, Tübingen, Germany). Reagent grade I water (18.2 MΩ cm; MilliQ IQ 7000, Merck-Millipore, Darmstadt, Germany) was used for all acid dilutions. Sample vials and pipette tips were pre-cleaned by soaking overnight in diluted sub-boiled nitric acid (*w* = 10% and subsequently *w* = 3% respectively) before use.

All standards were prepared in dilute sub-boiled acid (*w* = 2%). A Ca single element ICP-MS standard (*β* = 1000 µg mL^−1^; CertiPur, Merck, Darmstadt, Germany) was used as stock for calibration preparations. Single element standards of scandium (Sc, *β* = 1000 µg mL^−1^; Inorganic Ventures, Christiansburg, VA, USA), yttrium (Y, *β* = 10 µg mL^−1^; Elemental Scientific, Omaha, NE, USA), and indium (In, *β* = 1000 µg mL^−1^; CertiPur, Merck) were used to produce a mixed internal standard. Additional 1000 µg mL^−1^ single element ICP-MS standards of magnesium (*β* = 1000 µg mL^−1^; CertiPur, Merck) and potassium (*β* = 1000 µg mL^−1^; CertiPur, Merck) were used for interference testing.

### ICP-MS/MS measurements

All ICP-MS/MS measurements were carried out using a NexION 5000 (Perkin Elmer, Waltham, MA, USA), which is equipped with a dynamic reaction cell (DRC). Instrumental parameters are listed in Table 2. The sample was introduced to the ICP via a peristaltic pump. A mixed internal standard containing Sc (*w* = 4.7 ng g^−1^), Y (*w* = 2.2 ng g^−1^), and In (*w* = 2.2 ng g^−1^) was added online. Measurement parameters for the internal standards are displayed in Table 3. Calibrations in standard mode (both using single-quadrupole mode (Q3) and MS/MS mode) were carried out in the range of 5–1000 ng g^−1^ Ca and measured using the ^44^Ca isotope. DRC calibrations were carried out in the range of 0.2–100 ng g^−1^ Ca and measured using both the ^40^Ca and ^44^Ca isotopes. Using N_2_O, Ca was detected as the ^40^Ca^16^O^+^ and ^44^Ca^16^O^+^ product ions (mass shift of +16 amu).

**Table 2:** Instrument and plasma conditions for measurements made using ICP-MS/MS

Parameter	Q3	MS/MS	NH_3_ DRC MS/MS	N_2_O DRC MS/MS
Measurement mode	Standard	Standard	DRC on mass	DRC mass shift
Cell gas	None	None	NH_3_	N_2_O
Cell gas flow rate	None	None	0.7 mL min^−1^	0.4 mL min^−1^
RPa	0	0	0	0
RPq	0.25	0.25	0.45	0.45
Sample introduction	Peristaltic pump	Peristaltic pump	Peristaltic pump	Peristaltic pump
Nebulizer	PFA MicroFlow	PFA MicroFlow	PFA MicroFlow	PFA MicroFlow
Spray chamber	Peltier cooled SilQ cyclonic spray chamber	Peltier cooled SilQ cyclonic spray chamber	Peltier cooled SilQ cyclonic spray chamber	Peltier cooled SilQ cyclonic spray chamber
Spray chamber temperature	5 °C	5 °C	5 °C	5 °C
Interface cones	Nickel	Nickel	Nickel	Nickel
RF power	1600 W	1600 W	1600 W	1600 W
Ar nebulizer gas flow	0.98 L min^−1^	0.98 L min^−1^	0.98 L min^−1^	0.98 L min^−1^
Ar auxiliary gas flow	1.2 L min^−1^	1.2 L min^−1^	1.2 L min^−1^	1.2 L min^−1^
Ar plasma gas flow	16 L min^−1^	16 L min^−1^	16 L min^−1^	16 L min^−1^
QID fixed voltage	− 12 V	− 12 V	− 12 V	− 12 V
Hyperskimmer park voltage	5 V	5 V	5 V	5 V
OmniRing park voltage	− 185 V	− 185 V	− 185 V	− 185 V
Inner target lens voltage	2 V	2 V	2 V	2 V
Outer target lens voltage	− 7 V	− 7 V	− 7 V	− 7 V
Deflector exit voltage	− 8 V	− 8 V	− 8 V	− 8 V
Differential aperture voltage	− 3.5 V	− 3.5 V	− 3.5 V	− 3.5 V
Q1 AC rod offset	− 7 V	− 6 V	− 5 V	− 7.5 V
Q1 rod offset	− 11 V	− 2 V	− 0 V	0 V
Cell rod offset	− 33 V	− 33 V	− 5 V	− 2 V
Axial field voltage	0 V	0 V	150 V	250 V
Cell entrance voltage	− 10 V	− 5 V	− 6.5 V	− 7.5 V
Cell exit voltage	− 2 V	− 2 V	− 7 V	− 5 V
Q3 AC rod offset	− 2.5 V	− 2.5 V	− 8.5 V	− 8 V
Q3 rod offset	− 2 V	− 2 V	− 13 V	− 10 V
Dwell time	50 ms	50 ms	50 ms	50 ms
Scans	6	6	6	6
Replicates	6	6	6	6

**Table 3:** ICP-MS/MS measurement conditions for the tested internal standards using N_2_O and NH_3_ DRC

Internal standard	Reaction gas	Q1 *m*/*z*	Q3 *m*/*z*
Sc	N_2_O	45	61
Y	N_2_O	89	105
In	N_2_O	115	115
Sc	NH_3_	45	45
Y	NH_3_	89	89
In	NH_3_	115	115

### Cell gas flow rate optimization

Optimization of the reaction gas flow rates for NH_3_ and N_2_O was carried out on *m*/*z* 40 using a 10-ng g^−1^ single element Ca standard and a blank solution (*w* = 2% HNO_3_). Tested flow rates ranged from 0.1 to 1.0 mL min^−1^ using N_2_O and from 0.4 to 1.5 mL min^−1^ using NH_3_.

### Evaluation of matrix interferences

Evaluation of potential interference caused by Mg and K using N_2_O and NH_3_ DRC was carried out by measurement of the Ca background equivalence concentration in each respective single element standard at dilutions between 0.5 and 5.0 µg g^−1^. Determined Ca concentrations, made by means of external calibration, were used to evaluate the significance of each interference by comparing the mass fraction of Ca obtained using the non-interfered (by isobaric interference) isotope on *m*/z 44 (*w*(Ca)_*m/z*_ _44_) to that of the interfered isotope on *m*/z 40 (*w*(Ca)_*m/z*_ _40_). Where the difference was found to be significant, the magnitude of interference was calculated by subtraction of *w*(Ca)_*m/z*_ _44_ from *w*(Ca)_*m/z*_ _40_.

### Certified reference materials

River water certified reference materials (CRMs), SLRS-3 and SLRS-5 (both National Research Council Canada, Ontario, Canada), were used for validation. The materials were diluted to approximately 1 ng mL^−1^ Ca using dilute sub-boiled acid (*w* = 2% HNO_3_). Analysis was carried out using N_2_O and NH_3_ as reaction gases.

### Statistics

Statistical testing and the generation of figures were carried out using RStudio (version 2021.9.2.382). LOD and LOQ were calculated respectively from 3 and 10 times the standard deviation of 5 repeated measurements of a blank solution (*w* = 2% HNO_3_). Background equivalence concentration (BEC) was calculated from the calibration by division of the background (*y*-intercept) with the calibration slope. Equidistant standard concentrations of Ca between 0.2 and 1.0 ng g^−1^ were used when determining the LOD, LOQ, and BEC of the DRC methods.

Evaluation of the effect of interferences was carried out using total least squares (Deming) regression on the concentrations obtained using the ^40^Ca and ^44^Ca isotopes, where standard errors were calculated using the jackknife method. The observed *w*(Ca)_*m/z*_ _40_/*w*(Ca)_*m/z*_ _44_ ratio was statistically compared to the expected ratio of 1 using a *Z* test.

## Results and discussion

### Cell gas flow rate optimization

**Fig. 1:**
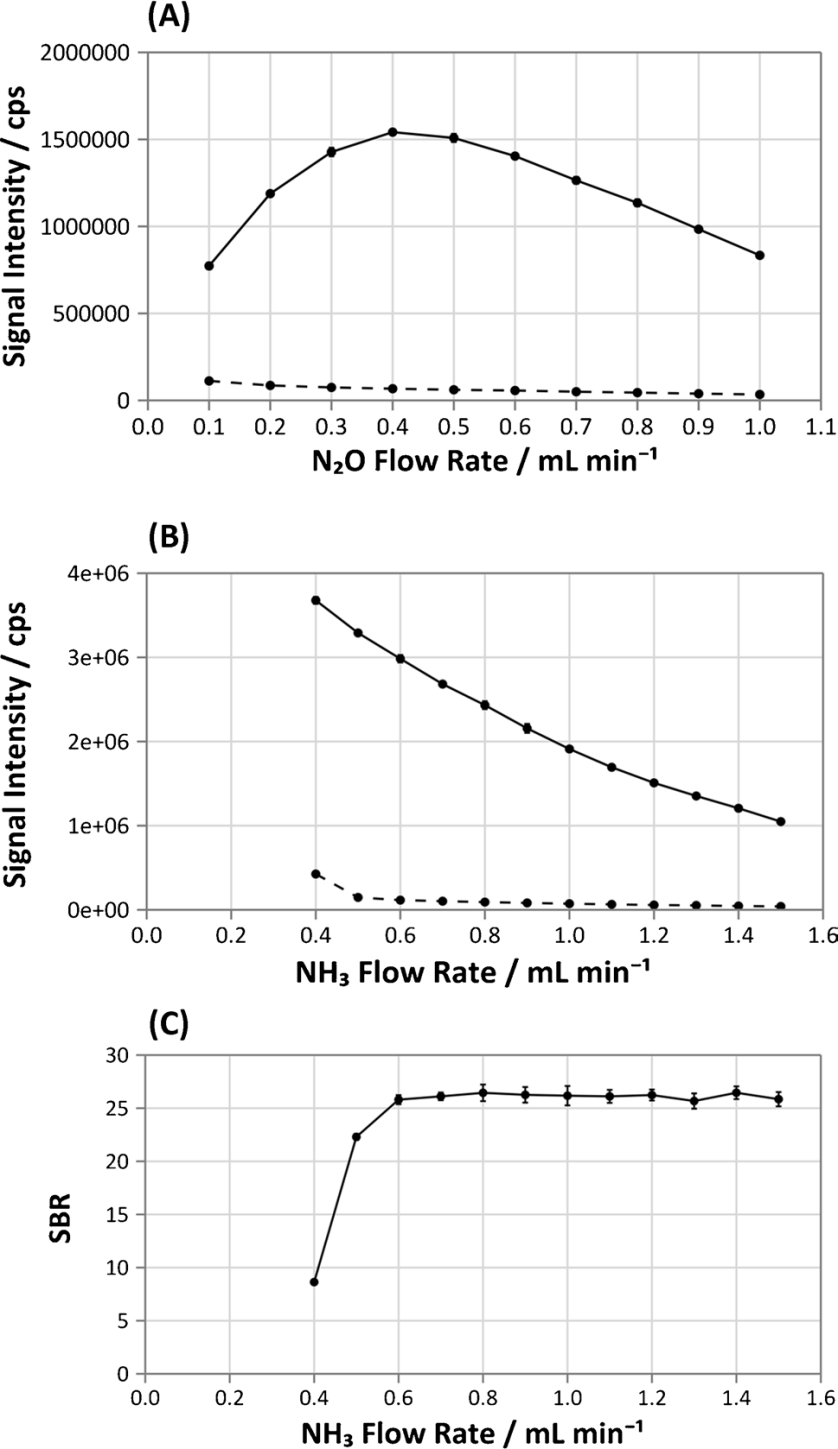
Optimization of sensitivity for 10 ng g^−1^ Ca (solid line) using **A** N_2_O and **B** NH_3_ cell gas flow rate for the removal of ^40^Ar interference on.^40^Ca. The background signal, measured using a blank solution, is also indicated (dotted line). Variation in the signal to background ratio (SBR) for NH_3_ DRC is shown in **C**. Error bars present represent one standard deviation of six replicates

The profile observed for mass shift using N_2_O (Fig. 1A) displayed optimum sensitivity for the formation of the ^40^Ca^16^O^+^ product ion at 0.4 mL min^−1^. The background signal of ^40^Ar^+^ was found to decrease as the N_2_O flow rate increased. Further observations of the blank signal conducted using the ^44^Ca isotope at optimum N_2_O conditions found a similar signal ratio of ^40^Ca/^44^Ca to that of the natural abundance. This suggests that, while a small degree of ^40^Ar^16^O^+^ forms at lower N_2_O flow rates, Ar was successfully removed by N_2_O at optimum conditions and the observed blank signal is primarily due to the presence of background Ca.

On-mass determination of Ca was carried out using NH_3_ flow rates between 0.4 and 1.5 mL min^−1^, as preliminary tests (carried out using ^38^Ar) suggested that lower flow rates would not remove enough Ar to prevent detector saturation on *m*/*z* 40. Within this range, the signal for Ca was observed to decrease with increasing NH_3_ flow (Fig. 1B). Consideration of the signal to background ratio demonstrated a plateau at NH_3_ flow rates greater than 0.7 mL min^−1^ (Fig. 1C); hence, this was determined to be the optimum condition. Similar to the use of N_2_O DRC, additional monitoring of the blank using ^44^Ca highlighted that the blank signal obtained on *m*/*z* 40 at the optimum NH_3_ flow rate was due to the presence of background Ca.

### Internal standards

**Fig. 2:**
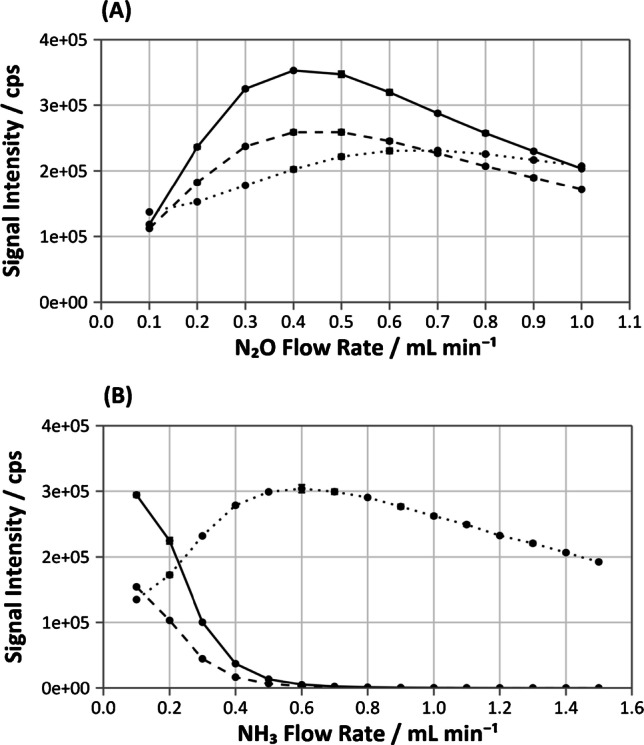
Variation of ^45^Sc (solid line), ^89^Y (dashed line), and.^115^In (dotted line) internal standard signal with **A** N_2_O and **B** NH_3_ cell gas flow rate. Error bars present represent one standard deviation of six replicates

Sc, Y, and In were tested as possible internal standards, as they have a narrow range of ionization energies (5.78–6.56 eV) close to that of Ca (6.11 eV) [18]. Variation of the internal standard response to the DRC gas flow rate is shown in Fig. 2. Initial tests using N_2_O DRC highlighted that In could not be measured in mass-shift mode, as no formation of the InO^+^ product ion was observed. However, detection of In^+^ on mass with N_2_O was found to be feasible, as an adequate and stable signal was obtained. Given that the sensitivity of the In signal increased with N_2_O flow rates up to 0.7 mL min^−1^, it could be interpreted that the signal observed may be enhanced by collisional focussing. Sc and Y, on the other hand, were observed to form the oxide product ion. Additionally, the sensitivity profiles closely matched that of Ca, with both internal standards displaying an optimum N_2_O flow rate of 0.4 mL min^−1^. The use of Sc and Y may then prove more advantageous in comparison to In, as they display similar cell reaction characteristics to that of the analyte.

In contrast, Sc and Y were observed to be unfit for use as internal standards when using NH_3_ as a reaction gas (Fig. 2B), as both elements were effectively removed when measuring on mass. The profile of In showed a collisional focussing effect, where a sufficient and stable signal was obtained for use as an internal standard at the previously determined optimum NH_3_ flow rate of 0.7 mL min^−1^. Given that, of the three internal standards considered, In was the only one found to be suitable for measurements, all subsequent data reported for NH_3_ DRC only includes internal normalization to In.

### Performance data

The instrument performance for the two DRC methods, as well as standard mode, was assessed and is presented in Table 4. Between the three internal standards used for N_2_O DRC, minimal differences in sensitivity (slope = 128,000–129,000 cps/ng g^−1^) and BEC (0.41–0.42 ng g^−1^) were observed. However, stability data indicated a lower RSD when using Sc (1.6%) as an internal standard compared to Y (2.2%) and In (2.9%). This was also reflected in the slightly lower LOD (of 0.015 ng g^−1^) and LOQ (of 0.049 ng g^−1^) values obtained using Sc. Therefore, while all three internal standards can be used for determination of Ca, Sc has been shown here to be the optimal in this case.

**Table 4:** Comparison of calibration parameters for the measurement of ^44^Ca with no reaction gas in Q3 and MS/MS mode, and.^40^Ca in MS/MS mode with N_2_O and NH_3_ reaction gas

Parameter	^44^Ca Q3 mode (no cell gas)	^44^Ca MS/MS mode (no cell gas)	^40^Ca on mass NH_3_ DRC	^40^Ca mass shift N_2_O DRC	^40^Ca mass shift N_2_O DRC	^40^Ca mass shift N_2_O DRC
Internal standard	Indium	Indium	Indium	Scandium	Yttrium	Indium
Slope (cps/ng g^−1^)	8260	3650	226,000	129,000	129,000	128,000
Intercept (cps)	150,000	56,500	82,500	52,600	52,700	53,300
Correlation, *R*	0.99983	0.99968	0.99832	0.99941	0.99924	0.99930
LOD (ng g^−1^)	0.14	0.27	0.015	0.015	0.017	0.017
LOQ (ng g^−1^)	0.43	0.81	0.049	0.049	0.057	0.056
BEC (ng g^−1^)	18	15	0.37	0.41	0.41	0.42
Stability test accuracy (%)*	–	–	101	101	99.3	97.0
Stability test RSD (%)*	–	–	2.39	1.57	2.20	2.92

^*^Based on 20 replicate measurements of a 0.5-ng g.^−1^ Ca standard

While the calibration slope observed for ^40^Ca using NH_3_ was 1.75 times greater than that of the N_2_O, the LOD (of 0.015 ng g^−1^) and LOQ (of 0.049 ng g^−1^) remained consistent. Additionally, determined BEC values (of 0.37 ng g^−1^ and 0.41 ng g^−1^ for NH_3_ and N_2_O DRC respectively) were similar between the two methods. This further indicates that the background signal for both DRC methods is likely due to background Ca levels. In comparison to the measurement of ^44^Ca using the Q3 mode with no cell gas (which is more sensitive than the MS/MS mode with no cell gas), the application of both NH_3_ and N_2_O DRC methods showed marked improvement, with approximately 10 times lower LOD (of 0.015 ng g^−1^) and LOQ (of 0.049 ng g^−1^), and approximately 45 times lower BEC (of 0.42 ng g^−1^).

### Removal of interferences

Mg and K were identified as the primary interferences of concern for measurements using the ^40^Ca isotope, as the polyatomic ^24^Mg^16^O^+^ interference formed in the plasma and isobaric interference from ^40^ K^+^ cannot be removed by the Q1 mass filter. Selenium, as ^80^Se^++^, was not considered to be of concern due to the high ionization energy required to form such interfering species [18] (Table 5).

**Table 5:** Total least squares regression results for the comparison of determined background Ca concentrations (*w* = 0–7.5 ng g^−1^) using isotopes ^44^Ca and ^40^Ca in high-concentration Mg (*w* = 0–4.0 µg g^−1^) and K (*w* = 0–4.4 µg g.^−1^) single element standards

DRC mode	Slope *w*(Ca)_*m/z*_ _40_ vs. *w*(Ca)_*m/z* 44_	Standard error of the slope	*Z* test *p* value	Significant?	Lower confidence interval	Upper confidence interval
Potassium interference						
N_2_O at 0.4 mL min^−1^ (Sc Int. Std.)	0.9917	0.0062	> 0.150	No	0.96518	1.01825
N_2_O at 0.4 mL min^−1^ (Y Int. Std.)	0.9916	0.0061	> 0.150	No	0.96532	1.01797
N_2_O at 0.4 mL min^−1^ (In Int. Std.)	0.9915	0.0061	> 0.150	No	0.96526	1.01777
NH_3_ at 0.7 mL min^−1^ (In Int. Std.)	1.1335	0.0022	< 0.001	Yes	1.12386	1.14314
Magnesium interference						
N_2_O at 0.4 mL min^−1^ (Sc Int. Std.)	0.9982	0.0107	> 0.150	No	0.95212	1.04429
N_2_O at 0.4 mL min^−1^ (Y Int. Std.)	0.9986	0.0111	> 0.150	No	0.95084	1.04626
N_2_O at 0.4 mL min^−1^ (In Int. Std.)	0.9987	0.0104	> 0.150	No	0.95399	1.04336
NH_3_ at 0.7 mL min^−1^ (In Int. Std.)	1.0062	0.0128	> 0.150	No	0.95135	1.06103

Native Ca concentrations in the measured single element standards of both Mg and K ranged between 1 and 10 ng g^−1^. For measurements of Ca with high Mg load, the *w*(Ca)_*m/z*_ _40_/*w*(Ca)_*m/z*_ _44_ ratio did not significantly differ from 1 for both N_2_O and NH_3_, which indicates that both gases can achieve successful removal of ^24^Mg^16^O^+^ interference on *m*/*z* 40. However, measurements of native Ca in high-level K standards using NH_3_ DRC showed a significantly greater *w*(Ca)_*m/z*_ _40_/*w*(Ca)_*m/z*_ _44_ ratio than expected, indicating significant bias introduced due to the presence of interfering ^40^ K that was not removed. Conversely, no such significant bias was observed for measurements of K single element standards using N_2_O DRC, suggesting that N_2_O can be used to successfully overcome the interference of K.

**Fig. 3:**
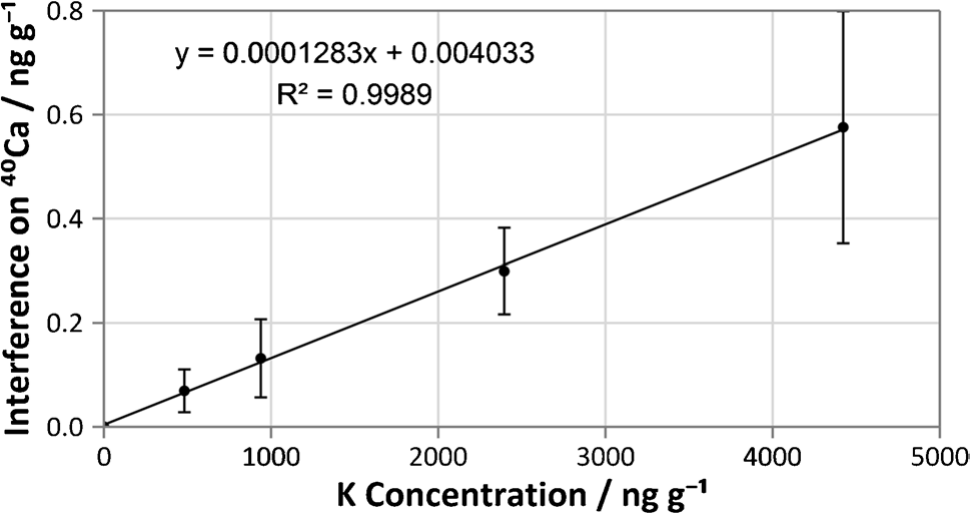
Contribution of ^40^ K interference on ^40^Ca measurements of single element K standards (*w* = 0–4.4 µg g.^−1^) using NH_3_ DRC. Error bars present represent the combined standard deviation of the difference between the observed *w*(Ca)_*m/z*_ _40_ and *w*(Ca)_*m/z*_ _44_ (calculated by the law of propagation of uncertainties)

**Fig. 4:**
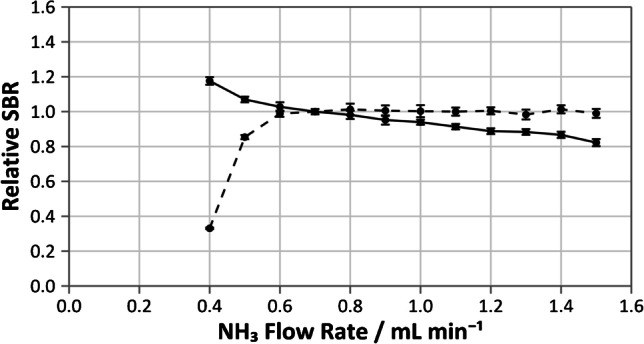
Variation of the relative signal to background ratio (SBR) with NH_3_ flow rate (normalized to the SBR observed at 0.7 mL min^−1^ NH_3_) for 10 ng g^−1 40^Ca signal with a 25 ng g^−1^.^39^ K background (solid line) and blank background on *m*/*z* 40 (dotted line). Error bars represent one combined standard deviation of six replicates (calculated by the law of propagation of uncertainties)

The level of interference from ^40^ K using NH_3_ DRC was determined by the difference of the observed *w*(Ca)_*m/z*_ _40_ and *w*(Ca)_*m/z*_ _44_ and plotted against the prepared K concentration (Fig. 3). The slope of the plot indicated that the level of interference on ^40^Ca appears as 0.0128% of the matrix K concentration. To obtain < 1% interference on ^40^Ca, matrix K concentrations cannot exceed about 80 times that of the analyte Ca concentrations. Further variation of the NH_3_ flow rate was not able to overcome the isobaric interference from K, as the Ca/K signal ratio only decreased with increasing flow rate (Fig. 4).

### Method validation

Validation of the total determination of Ca using N_2_O DRC was carried out using two river water CRMs that were certified for Ca. Results are shown in Table 6. Excellent recoveries between 99.2 and 103% were obtained for the two CRMs using Sc and Y as internal standards. Slightly lower recoveries of 95.4–96.9% were obtained using In as an internal standard, though these still fall within the stated uncertainty of the CRM. Recoveries of 97.9–98.5% were obtained using NH_3_ DRC, indicating that the N_2_O DRC method for determination of total Ca performs as well as existing methodology for real sample matrices.

**Table 6:** Measured mass fractions and recoveries of river water CRMs (diluted to 1 ng mL.^−1^) using N_2_O and NH_3_ DRC

CRM	Certified mass fraction (µg g^−1^)	N_2_O DRC (Sc internal standard)		N_2_O DRC (Y internal standard)		N_2_O DRC (In internal standard)		NH_3_ DRC (In internal standard)	
		Measured (µg g^−1^)	Recovery (%)	Measured (µg g^−1^)	Recovery (%)	Measured (µg g^−1^)	Recovery (%)	Measured (µg g^−1^)	Recovery (%)
SLRS-3	6.0 ± 0.4	6.09 ± 0.09	101	5.95 ± 0.10	99.2	5.72 ± 0.09	95.4	5.87 ± 0.09	97.9
SLRS-5	10.5 ± 0.4	10.8 ± 0.1	103	10.8 ± 0.1	103	10.2 ± 0.1	96.9	10.3 ± 0.1	98.5

## Conclusions

The data presented within this study suggests that N_2_O is not only a suitable replacement for NH_3_ for total Ca determinations, but can also ensure matrix-free determinations of ^40^Ca, especially in K-rich matrices with low Ca content. Despite apparent lower sensitivity of the N_2_O approach (by a factor of 1.75), detection limits and BEC were found to be similar between the two cell gases. We therefore propose that N_2_O should be used in place of NH_3_ for routine measurements of Ca, as well as incorporating this approach into wider multi-element analysis.
